# The Story of the Insane from Year to Year

**Published:** 1899-09-23

**Authors:** 


### THE STORY OF THE INSANE FROM YEAR TO YEAR.

(Eleventh Series.)

### THE NORTH RIDING ASYLUM AT YORK.

There were 751 patients in residence at the beginning of the year, and by the last day of December the number had risen to 781. There were 170 first admissions, 32 readmissions, 86 recoveries, and 77 deaths. The recoveries stand at the very creditable percentage of 49·21 of the admissions, which is considerably above the average at this asylum, and also above the county asylums average generally. The deaths stand at 10·11 per cent. on the average number resident. Twenty-nine of the deaths were caused by cerebral and spinal diseases, 11 by consumption, 5 by pneumonia, and 5 by pleurisy. Intemperance in drink accounts for 34 of the year's admissions, hereditary influence for 35, while various moral causes, such as domestic trouble and adverse circumstances, account for 50. Dr. Hingston mentions the case of a criminal lunatic "of the worst type, for whom the ordinary appliances of an asylum were totally inadequate for his safe detention." This madman escaped on three occasions and committed violent outrages. At last the Home Secretary gave an order for his removal to Broadmoor. Most of our county asylums have, or have had, parallel cases; but the staff has to endure all the risk attending these patients, and there is no hope of getting rid of them until an assault is committed. Our modern county asylums were not intended for the reception of such cases, and united action should be taken in the matter before some serious accident happens. The weekly rate of maintenance is 9s. 7½d. per head.

### LANCASHIRE COUNTY ASYLUM AT WHITTINGHAM.

On January 1st, 1897, this asylum contained the enormous number of 1,938 patients, and at the end of the year the numbers were practically stationary. There were 330 first admissions, 24 readmissions, 145 recoveries, and 173 deaths. Put into percentages these numbers show that the recoveries reached 41·07 on the admissions, and the deaths 8·91 on the average number resident. Of these deaths 58 were caused by cerebral and spinal diseases, 36 by consumption, and 9 by enteric fever. Intemperance in drink is the assigned cause of the insanity in 112 of the year's admissions, hereditary tendency in 51, and previous attacks in 57. In the treatment of diseases Dr. Percival and his staff have had an experience more varied than pleasant, for, in addition to those already mentioned as ending in death, they had 106 cases of influenza, 8 of erysipelas, 5 of typhoid fever, and 28 cases of scarlet fever. The lectures to the attendants have been continued during the year, and Dr. Percival says that the "intelligent interest and industry displayed by the attendants has been very gratifying." Unquestionably the more an attendant knows about his work the better he will perform that work. The average cost per head per week was 8s. 6¾d.

### WILTS COUNTY ASYLUM, DEVIZES.

This asylum contained 789 patients on January 1st, 1897, and 825 on the last day of December. There were 136 admissions, 22 readmissions, 57 recoveries, and 56 deaths. The recoveries stand at 36 per cent. on the admissions, and the deaths at 6·8 per cent. on the average number resident. Of the deaths 12 were owing to cerebral and spinal diseases, 6 to phthisis, and 9 to pneumonia. Table X., which shows the causes of insanity in the year's admissions, tells us that in 30 cases the cause was of moral nature, such as adverse circumstances and mental anxiety; in 13 it was intemperance in drink, and in 45 it was hereditary tendency. Commissioners in Lunacy and medical superintendents have lately had their minds much exercised on the question of the real or supposed increase of insanity. Mr. Bowes feels satisfied that, so far as his own county is concerned, there has been no increase in the disease—that is, no relative increase—and he seems to prove it. This is consolatory so far as it goes, but, unfortunately, Mr. Bowes has to add that there will be a further accumulation of the insane, and he proves this too. The asylum is to be considerably added to, and when the additions are finished it will contain 950 beds, beyond which it is to be hoped it will never be allowed to go. Lectures are given by Dr. Barraclough to those nurses who wish to take the certificate granted by the Medico-Psychological Association. This is a good step to take, and we hope that neither Dr. Barraclough nor the nurses will rest satisfied with this certificate, but that Mr. Bowes will be induced to grant a "Three years' Devizes certificate," and so bring his nurses up to the hospital level. The committee say that the "management of the asylum in every respect has their highest approbation." The average weekly cost of maintenance was 8s. 9d. per head.

